# 不同家系遗传性蛋白C缺陷症12例临床表型和基因突变分析

**DOI:** 10.3760/cma.j.issn.0253-2727.2022.01.008

**Published:** 2022-01

**Authors:** 琦煜 徐, 丽红 杨, 海啸 谢, 艳慧 金, 小龙 李, 星星 周, 媚娜 刘, 明山 王

**Affiliations:** 温州医科大学附属第一医院医学检验中心，温州 325015 Department of Clinical Laboratory, The First Affiliated Hospital of Wenzhou Medical University, Wenzhou 325015, China

**Keywords:** PROC基因, 凝血因子PC缺陷症, 基因突变, 静脉血栓形成, PROC gene, Protein C deficiency, Genetic mutation, Venous thrombosis

## Abstract

**目的:**

探讨来自不同家系12例遗传性蛋白C（PC）缺陷症先证者的基因突变类型与临床特征。

**方法:**

采用发色底物法检测血浆PC活性，酶联免疫吸附法检测PC抗原含量。采用PCR直接测序法分析先证者PROC基因9个外显子及其侧翼序列，对发现的疑似突变用反向（缺失突变用克隆）测序予以验证。

**结果:**

12例先证者的PC活性均明显下降（18％～55％），其中10例先证者的PC抗原水平显著降低（13％～58％）。共发现11种PROC基因突变，其中c.383G>A（p.Gly128Asp）、c.997G>A（p.Ala291Thr）、c.1318C>T（p.Arg398Cys）和c.532G>C（p.Leu278Pro）4种杂合突变为首次发现；6种突变发生在丝氨酸蛋白酶结构域、4种发生在表皮生长因子同源区域（EGF）、1种突变在EGF和丝氨酸蛋白酶结构域之间的激活肽区域；缺失突变（p.Met364Trp fsX15和p.Lys192del）2种，其余为错义突变。有3例无亲缘关系的先证者检出p.Phe181Val和p.Arg189Trp纯合或杂合突变。所有基因突变可能来自先证者的父亲和（或）母亲，其中2个家系存在近亲婚配。先证者有9例出现静脉血栓形成、2例有不良妊娠表现、1例出现紫癜。

**结论:**

PROC基因缺陷导致的PC缺陷症患者易发生静脉血栓形成，尤其当同时存在其他易栓因素时。

## Abstract (en)

**Objective:**

To investigate the molecular pathogenesis and clinical features of unrelated 12 patients with inherited coagulation protein C（PC）deficiency in Chinese population.

**Methods:**

The PC activity（PC:A）and PC antigen（PC:Ag）were detected by chromogenic substrate and enzyme linked immunosorbent assay, respectively. The nine exons and flanking sequences of the protein C（PROC）gene were amplified by polymerase chain reaction with direct sequencing, and the suspected mutations were validated by reverse sequencing（clone sequencing for deletion mutations）.

**Results:**

The PC:A of the 12 probands decreased significantly, ranging from 18% to 55%, and the PC:Ag of the 10 probands decreased significantly. Eleven mutations were found, out of which four mutations［c.383G>A（p.Gly128Asp）, c.997G>A（p.Ala291Thr）, c.1318C>T（p.Arg398Cys）, and c.532G>C（p.Leu278Pro）］were discovered for the first time. Six mutations were in the serine protease domain, four mutations were located in epidermal growth factor（EGF）-like domains, and one mutation was located in activation peptide. There were two deletion mutations（p.Met364Trp fsX15 and p.Lys192del）, and the rest were missense mutations. Mutations p.Phe181Val and p.Arg189Trp were identified in three unrelated families. All mutations may be inherited, and consanguineous marriages were reported in two families. Among the probands, nine cases had venous thrombosis, two cases had poor pregnancy manifestations, and one case had purpura.

**Conclusion:**

Patients with PC deficiency caused by PROC gene defects are prone to venous thrombosis, especially when there are other thrombotic factors present at the same time.

蛋白C（PC）是由肝脏合成的一种维生素K依赖性糖蛋白，为机体PC/蛋白S（PS）抗凝系统中的重要组成部分，在钙离子和磷脂的参与下，PC被内皮细胞表面的凝血酶-血栓调节蛋白复合物激活成活化蛋白C（APC）[1]。APC具有抗凝、促纤溶和维持血管内皮屏障稳定性等功能[2]。PC缺乏患者的临床表现具有高度多样性，并且是深静脉血栓形成（DVT）和肺血栓栓塞症的高危人群[3]。遗传性PC缺陷症通常为常染色体显性遗传，是一种由编码蛋白C的基因（PROC）突变导致PC含量或（和）功能异常的遗传性疾病。本研究对12例来自不同家系遗传性PC缺陷症患者的PROC基因和临床表现进行分析，并探讨其分子发病机制与临床特征的关系。

## 病例与方法

1. 病例资料：先证者1，女，43岁，因“左下肢疼痛27 d”就诊，B超显示“左下肢DVT”，8岁开始四肢关节活动受限、疼痛，平时间断使用“丹参”和“布洛芬”，育龄期长期口服避孕药，父母为姨表近亲婚配，其弟弟10岁始有与先证者相同症状。先证者2，男，8岁，因紫癜待查入院，血细胞计数检查血小板数量无异常，凝血功能检查结果仅PC水平显著降低。先证者3，男，55岁，左下肢出现发绀伴疼痛，B超检查提示“左侧髂外DVT”，其父亲因“脑静脉窦血栓”而死亡。先证者4，女，32岁，不明原因停胎2次就诊。先证者5，男，25岁，左下肢疼痛1周，B超显示“左下肢DVT”。先证者6，女，39岁，因下肢疼痛伴头痛就诊，B超示“左下肢DVT”，头颅MRI显示颅内静脉窦血栓形成（CVST），同时患有系统性红斑狼疮（SLE），其父亲死于“脑静脉窦血栓形成”，哥哥因肺静脉栓塞而死亡。先证者7，女，27岁，不明原因停胎2次就诊。先证者8，男，53岁，因左下肢疼痛就诊，B超显示“左下肢DVT”，有长期吸烟史。先证者9，男，17岁，持续性头痛就诊，头颅CT血管造影显示CVST，有长期制动生活习惯。先证者10，男，28岁，因咳嗽，痰中带血就诊，胸部CT血管造影显示“肺栓塞”。先证者11，女，30岁，因突发左侧肢体无力伴四肢抽搐就诊，头颅MRI提示CVST（上矢状窦），其大舅和二舅分别有脑梗死病史和下肢DVT病史。先证者12，女，46岁，因头痛就诊，头颅CT显示CVST，父母为姑表近亲婚配。

150名正常对照为我院健康体检者，男81名，女69名，中位年龄32（18～60）岁。对照组经查均无肝肾功能疾病，无出血和血栓史，研究通过本院伦理委员会审查（2012-17）。

2. 标本采集：采集受试者外周静脉血2.7 ml于含0.109 mol/L枸橼酸钠抗凝管9∶1抗凝，3 000 r/min离心10 min。上清乏血小板血浆用于表型指标检测，2 h内检测完毕；下层血细胞用于提取基因组DNA，−40 °C冻存待检。基因组DNA提取试剂盒购自于北京天根生化科技有限公司，提取步骤按照说明书进行。

3. 凝血指标检测：在法国Stago STA-R全自动血凝仪上使用仪器配套试剂盒用发色底物法检测PC活性（PC∶A）和抗凝血酶活性（AT∶A）；凝固法检测蛋白S活性（PS∶A）。PC抗原（PC∶Ag）采用酶联免疫吸附法（ELISA）检测，试剂盒购自温州长风生物技术有限公司。

4. DNA提取及PCR扩增：在ABI Thermal cycler 2720扩增仪上扩增PROC基因所有9个外显子及侧翼序列，所用引物序列及实验过程参照文献[4]。整个PCR反应体系为25 µl，包括2×Taq PCR MasterMix（购自北京天根生化科技有限公司）12.5 µl，去离子水7.5 µl，模板DNA 3 µl以及引物2 µl（正向引物和反向引物各1 µl）。反应条件：95 °C预变性5 min；95 °C变性30 s，根据不同引物分别以相应退火温度（58～64 °C）退火30 s，72 °C延伸30 s，共30个循环；最后72 °C延伸10 min；PCR扩增产物于4 °C保存。

5. DNA序列及分析：用GoldviewI做标志物，经1.5％琼脂糖凝胶电泳鉴定为阳性的PCR产物送上海派森诺生物科技股份有限公司进行纯化后直接测序。测序结果用Chromas软件与美国NCBI基因文库中的PROC基因序列进行比对，寻找基因突变位点。发现基因突变的序列则反向测序予以证实，疑似插入突变的序列则用克隆测序予以证实。对150名健康对照组人群相应的区域进行PCR扩增、测序，用于排除基因多态性。

6. pMD18-TTA克隆载体：将含有缺失突变的序列克隆入pMD18-TTA克隆载体中，分别对克隆入载体的单条染色体上的 PROC 基因序列进行测序。

## 结果

1. 血浆表型检测结果：所有先证者的PC∶A均呈不同程度降低，除先证者8、12的PC∶Ag正常外，其余10例PC∶Ag均呈同步下降。所有先证者的PS∶A、AT∶A均在正常范围内。12例先证者的PC水平及临床特征见表1。

**表1:** 12例遗传性蛋白C（PC）缺陷症先证者的PC活性（PC∶A）、PC抗原（PC∶Ag）水平及临床表现

例号	性别	年龄（岁）	PC∶A（％）	PC∶Ag（％）	分型	临床表现	其他易栓诱因
1	女	43	20	13	Ⅰ型	下肢DVT	口服避孕药
2	男	8	18	58	Ⅰ型	紫癜	无
3	男	55	35	44	Ⅰ型	下肢DVT	高龄
4	女	32	46	50	Ⅰ型	复发性流产	妊娠
5	男	25	40	53	Ⅰ型	下肢DVT	无
6	女	39	35	45	Ⅰ型	下肢DVT、CVST	SLE
7	女	27	55	53	Ⅰ型	复发性流产	妊娠
8	男	53	50	75	Ⅱ型	下肢DVT	吸烟
9	男	17	40	51	Ⅰ型	CVST	制动
10	男	28	23	57	Ⅰ型	肺栓塞	无
11	女	30	26	19	Ⅰ型	CVST	无
12	女	46	21	78	Ⅱ型	CVST	无

注：DVT：深静脉血栓形成；CVST：颅内静脉窦血栓形成；SLE：系统性红斑狼疮。参考值：PC∶A 70％～130％，PC∶Ag 70％～140％

2. PROC基因突变特性：在12例遗传性PC缺陷症先证者中共发现11种类型的15个基因突变（表2、图1），其中c.383G>A（p.Gly128Asp）、c.997G>A（p.Ala291Thr）、c.1318C>T（p.Arg398Cys）和c.532G>C（p.Leu278Pro）4种突变为首次发现，150名健康对照者相同位点未发现上述突变，排除基因多态性的可能；11种基因突变中2种为缺失突变c.1212-1212delG（p.Met364Trp fsX15）和c.577-579delAAG（p.Lys192del），余9种均为错义突变；有6种突变发生在丝氨酸蛋白酶结构域、4种发生在EGF区域、1种突变在EGF和丝氨酸蛋白酶结构域之间的激活肽区域；各有3例先证者存在p.Phe181Val和p.Arg189Trp纯合或杂合突变，这两种突变在无亲缘关系的家系中重复出现。先证者1、12分别为p.Phe181Val和p.Arg189Trp纯合突变，先证者3、4、5、6、7、8、9分别为p.Leu278Pro、p.Met364Trp fsX15、p.Ala251Val、p.Ala291Thr、p.Arg398Cys、p.Arg189Trp和p.Ala178Pro杂合突变，先证者2、10、11分别为p.Gly128Asp和p.Arg189Trp、p.Lys192del和p.Phe181Val、p.Asp297His和p.Phe181Val双杂合突变；通过对家系成员分析，推测先证者的基因突变可能遗传自其父亲和（或）母亲，其中2个家系（先证者1、12）父母存在近亲婚配关系。

**表2:** 12例遗传性蛋白C缺陷症患者的PROC基因检测结果

例号	核苷酸突变	氨基酸变化	外显子	功能区	突变类型
1	c.6128T>G	p.Phe181Val	7	EGF	纯合
2	c.383G>A	p.Gly128Asp	5	EGF	杂合
	c.565C>T	p.Arg189Trp	7	激活肽	杂合
3	c.833T>C	p.Leu278Pro	9	丝氨酸蛋白酶结构域	杂合
4	c.1212-1212delG	p.Met364Trp fsX15	9	丝氨酸蛋白酶结构域	杂合
5	c.752C>T	p.Ala251Val	9	丝氨酸蛋白酶结构域	杂合
6	c.997G>A	p.Ala291Thr	9	丝氨酸蛋白酶结构域	杂合
7	c.1318C>T	p.Arg398Cys	9	丝氨酸蛋白酶结构域	杂合
8	c.565C>T	p.Arg189Trp	7	激活肽	杂合
9	c.532G>C	p.Ala178Pro	5	EGF	杂合
10	c.577_-_579delAAG	p.Lys192del	7	EGF	杂合
	c.541T>G	p.Phe181Val	7	EGF	杂合
11	c.8478G>C	p.Asp297His	9	丝氨酸蛋白酶结构域	杂合
	c.6128T>G	p.Phe181Val	7	EGF	杂合
12	c.565C>T	p.Arg189Trp	7	激活肽	纯合

注：EGF：表皮生长因子同源区域

**图1:**
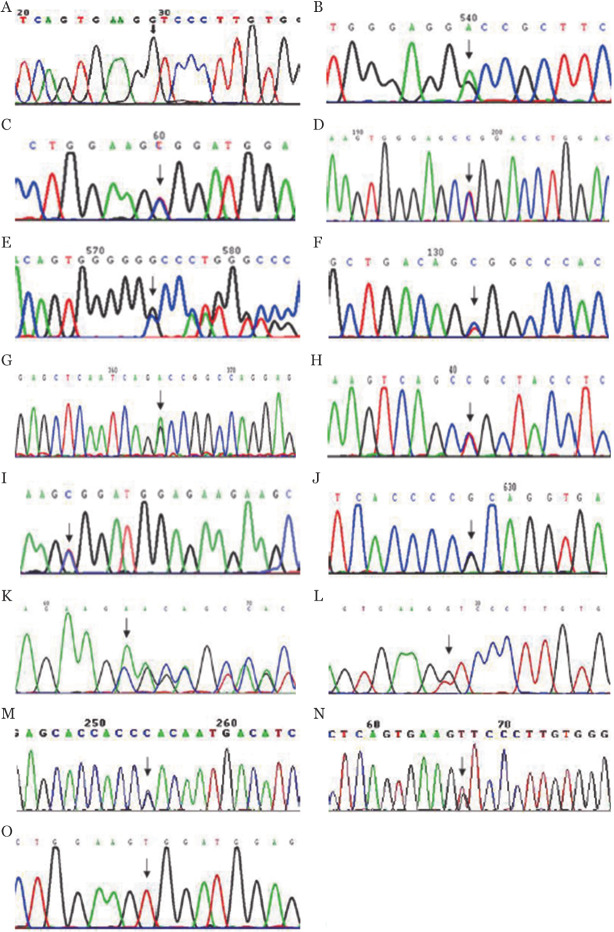
12例遗传性蛋白C缺陷症患者PROC基因测序结果 A：先证者1 PROC基因7号外显子c.6128T>G纯合突变；B、C：先证者2 PROC基因5号外显子c.383G>A和7号外显子c.565C>T杂合突变；D：先证者3 PROC基因9号外显子c.833T>C杂合突变；E：先证者4 PROC基因9号外显子c.1212-1212delG杂合突变；F：先证者5 PROC基因9号外显子c.752C>T杂合突变；G：先证者6 PROC基因9号外显子c.997G>A杂合突变；H：先证者7 PROC基因9号外显子c.1318C>T杂合突变；I：先证者8 PROC基因7号外显子c.565C>T杂合突变；J：先证者9 PROC基因5号外显子c.532G>C杂合突变；K、L：先证者10 PROC基因7号外显子c.577-579delAAG和c.541T>G杂合突变；M、N：先证者11 PROC基因9号外显子c.8478G>C和7号外显子c.6128T>G杂合突变；O：先证者12 PROC基因7号外显子c.565C>T纯合突变

## 讨论

人类PROC基因定位于染色体2q13-q14，全长11.2kb，由9个外显子组成，分别编码羧基谷氨酸（Gla）结构域、EGF、连接肽、激活肽和丝氨酸蛋白酶样结构域，其中丝氨酸蛋白酶样结构域为蛋白C的活化中心 [5]。遗传性PC缺陷症分为两种类型[6]：Ⅰ型为PC∶A和PC∶Ag同步降低，Ⅱ型为PC∶A降低、PC∶Ag正常，约75％的PC缺乏症患者为Ⅰ型。遗传性PC缺陷症主要与PROC基因突变有关，1981年由Griffin等首次报道[7]。由于PROC基因的突变导致PC产生、聚合、分泌及功能受影响。

截至目前，人类基因突变数据库（HGMD）共列出了400多个PROC基因突变，其中错义/无义突变占主要部分，小部分为插入/缺失突变和剪接突变。本组12例遗传性PC缺陷症患者检出11种类型的基因突变，其中5号外显子c.383G>A（p.Gly128Asp）、9号外显子c.997G>A（p.Ala291Thr）、c.1318C>T（p.Arg398Cys）和c.532G>C（p.Leu278Pro）共4种突变为新发现的突变位点。突变类型包括错义突变（9种）和缺失突变（2种），其中6种突变发生在丝氨酸蛋白酶结构域、4种发生在EGF区域，可见这些区域可能是本地区PROC基因较易发生突变及导致蛋白C功能异常的主要区域，1种突变在EGF和丝氨酸蛋白酶结构域之间的激活肽区域。并且还发现了本地区人群中存在导致遗传性PC缺陷的突变热点：各有3例存在p.Arg189Trp和p.Phe181Val纯合或杂合突变在无亲缘关系的家系中重复出现。一些突变位点（p.Arg272Cys、p.Arg220Trp、p.Gln174X、p.Val339Met和p.Pro210Leu）一般多见于高加索人，然而其他一些突变位点（p.Phe181Val、p.Arg211Trp、p.Val339Met、p.Met406Ile和p.G8857del）在日本人中常见[8]。Ding等[9]发现p.Arg189Trp及p.Lys192del是导致中国人群PROC基因缺陷的主要突变类型。此外，p.Arg189Trp也是中国台湾人群中最常见的突变类型[10]。这些研究结果表明PROC基因突变受种族的影响，亚洲地区与欧洲地区PROC基因突变类型有很大不同。

PC缺陷症常导致血栓形成，通常表现为静脉血栓，本组病例以DVT和CVST常见。Gu等[11]在202例中国静脉血栓患者中发现PC缺陷症患病率约为8％。根据突变基因型不同，可分为纯合子型和杂合子型（单杂合子型和复合杂合子型）；纯合子型和复合杂合子型为严重遗传性PC缺陷，PC∶A非常低；大部分遗传性PC缺陷症患者为单一位点杂合突变所致，PC检测水平大约为正常值的50％左右，一般无临床症状或成年期才发生静脉血栓栓塞[12]。朱铁楠等[13]研究发现，PC∶A在不同性别中国汉族人群中随年龄增长而增加，但在50岁以上男性人群中呈下降趋势。本研究中先证者1、12为纯合突变，先证者2、10、11为复合杂合突变，PC∶A均明显低于单杂合子型患者，且在无明显其他易栓诱因情况下即有血栓形成表现。PC缺陷症患者发生血栓的危险性随年龄增高而增加，是否出现血栓形成临床症状还与多种环境危险因素（妊娠、外伤、服用避孕药、吸烟、制动等）及基因突变共同作用有关。本研究中，先证者1于8岁开始发生四肢关节活动受限、疼痛，成年后又长期口服避孕药；先证者3为高龄患者；先证者4、7为妊娠妇女；先证者6患有SLE；先证者8有吸烟嗜好；先证者9有制动生活习惯。这些因素均可能是患者静脉血栓形成的发病诱因。

与PC功能密切相关的位点突变对PC水平影响较大，其中p.Phe181Val[14]、p.Asp297His[15]、p.Met364Trp fsX15[16]、p.Gly128Asp[4]和p.Ala178Pro[17]通过构建蛋白模型或体外表达研究，发现蛋白质结构改变或分泌障碍或细胞内部分降解加速，是导致血浆中PC含量降低或活性异常的主要原因。p.Ala251Val和p.Ala291Thr位于丝氨酸蛋白酶样结构域，氨基酸取代可能会损害重链的结构完整性和稳定性，并削弱蛋白质的催化活性，导致PC∶A降低[18]–[19]。在本研究中，先证者11两个突变基因所在区域功能不同，可能对PC∶A的影响也不同，但两个突变的相互作用可能会加重这种影响。有研究报道，p.Arg189Trp[4]和p.Lys192del[20]–[21]在激活位点及Gla结构域中具有正常功能，对PC的分泌和降解并没有显著影响，但在与PS或活化凝血因子Ⅴ和Ⅷ相互作用的功能中存在缺陷，其导致的结构变化，会阻碍PC与细胞表面、磷脂、PS以及部分凝血因子的作用，因此表现为Ⅱ型PC缺陷症（PC∶Ag含量正常）。p.Arg189Trp和p.Lys192del二者可分别使血栓栓塞的发生风险增加5～7倍和2～3倍[22]。p.Arg398Cys和p.Leu278Pro对PC功能的影响将有待于进一步的研究。

综上所述，我们对12例遗传性PC缺陷症患者的基因突变和临床特征进行了分析，发现本地区人群中存在导致遗传性PC缺陷的突变热点p.Arg189Trp和p.Phe181Val。通过调查先证者家系情况及临床表现，表明PROC基因缺陷导致的PC缺陷症患者易发生静脉血栓形成，尤其当同时存在其他易栓因素时。
